# Optical signatures of radiofrequency ablation in biological tissues

**DOI:** 10.1038/s41598-021-85653-0

**Published:** 2021-03-22

**Authors:** Pranav Lanka, Kalloor Joseph Francis, Hindrik Kruit, Andrea Farina, Rinaldo Cubeddu, Sanathana Konugolu Venkata Sekar, Srirang Manohar, Antonio Pifferi

**Affiliations:** 1 Department of Physics, Politecnico di Milano, Milan, Italy; 2 Multi-Modality Medical Imaging Group, University of Twente, Enschede, The Netherlands; 3 Biomedical Photonic Imaging Group Technical Medical Centre, University of Twente, Enschede, The Netherlands; 4 Institute of Photonics and Nanotechnologies, National Research Council, Milan, Italy; 5 Biophotonics@Tyndall, IPIC, Tyndall National Institute, Lee Maltings, Dyke Parade, Cork, Ireland

**Keywords:** Near-infrared spectroscopy, Diagnostic markers

## Abstract

Accurate monitoring of treatment is crucial in minimally-invasive radiofrequency ablation in oncology and cardiovascular disease. We investigated alterations in optical properties of ex-vivo bovine tissues of the liver, heart, muscle, and brain, undergoing the treatment. Time-domain diffuse optical spectroscopy was used, which enabled us to disentangle and quantify absorption and reduced scattering spectra. In addition to the well-known global (1) decrease in absorption, and (2) increase in reduced scattering, we uncovered new features based on sensitive detection of spectral changes. These absorption spectrum features are: (3) emergence of a peak around 840 nm, (4) redshift of the 760 nm deoxyhemoglobin peak, and (5) blueshift of the 970 nm water peak. Treatment temperatures above 100 °C led to (6) increased absorption at shorter wavelengths, and (7) further decrease in reduced scattering. This optical behavior provides new insights into tissue response to thermal treatment and sets the stage for optical monitoring of radiofrequency ablation.

## Introduction

The use of radiofrequency currents to induce heat in tissue is known for over a century from the time of D’Arsonval^1^. The prevailing form of percutaneous radiofrequency ablation (RFA) was introduced in 1990 by McGahan et al.^2^, as a treatment option when open surgery is not viable. RFA is based on delivering a high-frequency alternating current into the tissue using a slender needle device with electrodes. The current flows into the tissue from the device to a ground plate located usually on the back or chest of the subject. The flow of current causes ionic agitation in tissue underlying the device’s electrode where the current density is highest, which generates frictional or Joule heating in the region of the tissue around the electrode, which then conducts through the rest of the tissue. The temperature rise causes irreversible changes such as protein denaturation, melting of the cellular lipid bilayer and evaporation of intracellular fluid leading to coagulative necrosis^3^. RFA is in clinical use for the treatment of tumors in the liver, lungs, kidneys, brain and bones, to remove abnormal electrical paths in cardiac arrhythmia, for pain management in the musculoskeletal system especially for the lumbar region and in the knee, and for treating several other diseases^4–6^. The last decade has experienced rapid growth in the clinical use of RFA with high demand for minimally invasive treatment for a better quality of life^7^. However, with the possibility of incomplete ablation of the target tissue, there is a high chance of recurrence of the disease. A study on liver cancer treatment with RFA reported a recurrence rate as high as 47%^8^, and about 20–40% of the patients treated for atrial fibrillation underwent a second ablation^9^. Incomplete ablation could primarily be attributed to two factors. The first is a cooling of the ablation region by large blood vessels known as the heat sink effect, and the second is carbonization around the electrode due to desiccation at high temperature causing rapid increase in electrical impedance, inhibiting current flow and further heating^10^. To overcome these issues there is a critical need to accurately monitor the RFA procedure in real-time and to get reliable markers differentiating the degree of thermal damage experienced by the tissue at a given point of time during the treatment. Accurate RFA treatment will spread its use resulting in significant improvement in life quality for the patient as compared to open-chest surgery, and reduction in health-care costs. Temperature measurement at the tip of the electrodes or tissue impedance are generally used as indicators of the thermal damage occurring in the tissue, but these measurements provide limited information on the ablation size and the location of the boundary. Monitoring of the ablation procedure is therefore also guided using imaging techniques based on ultrasound (US), computed tomography (CT) or magnetic resonance (MR). However, using US to monitor the ablation zone is challenging because of the RFA generated hyperechogenic response^11^. Unenhanced US is therefore primarily used for estimating the ablation volume. CT-guided RFA is also limited for delineating the induced ablation zone. Guidance of RFA using magnetic resonance imaging (MRI) outperforms both these imaging modalities but has the drawback of increased treatment complexity and cost^12,13^.

Thus, there is a definite need for alternative monitoring techniques which are independent of the thermal disturbances occurring in the tissue during treatment. Moreover, such techniques should be non-invasive with the possibility of real-time operation to facilitate immediate feedback in a clinical scenario. Diagnostic or monitoring tools that rely on the optical characteristics of the treated tissue are potentially good candidates in this direction. Optical techniques employing parameters such as diffuse reflectance and transmittance^14–16^, fluorescence^14^, absorbance using visible light spectroscopy^17^ and optical properties^18–24^ (absorption and scattering coefficient), have obtained interesting initial results in studying the effect of thermal treatment on biological tissue^25^. Many distinct transformations occur during RFA treatment, involving both microstructure alterations leading to scattering changes, and modification in different tissue constituents (e.g. protein denaturation) leading to absorption changes. The above-mentioned studies used Continuous Wave (CW) diffuse optical spectroscopy (DOS) techniques^15–24^, which have the advantages of being relatively inexpensive and easily scalable^26^. Yet CW approaches rely on the attenuation of light by the tissue which is caused by the tightly coupled effect of both absorption and scattering as well as optical injection and collection coupling at the fiber tips, which can be severely altered during treatment. Thus, CW monitoring measures optical signals which are a complex result of several factors, not easy to disentangle during operation. This results in an incomplete extraction of the optical properties of the interrogated tissue which potentially holds the key to the accurate monitoring of RFA therapies.

An alternative option is the adoption of a Time-Domain (TD) approach, based on illumination with short (ps) laser pulses and detection of the Distribution of Time-Of-Flight (DTOF) of randomly scattered photons. TD based DOS instrumentation has the advantage to naturally disentangle the absorption from the scattering coefficient out of a single measurement^27^ since both parameters have quite distinct effects on the DTOF. This capacity to decouple the two optical properties can be of great value to separate distinct thermal effects, leading to identification of specific markers to grade tissue transformation during RFA treatment. Further, the approach is unaffected by factors like signal fluctuations, skin pigmentation, optical contact with the sample, surface roughness and irregularities^25^, since it relies only on the temporal shape of the DTOF and not on its amplitude. Thus, we expect negligible artefacts due to bleeding, charring at the fiber tip or bubble formation often related to RFA treatment^28^. Also, it provides the absolute estimate of the absorption and scattering coefficient without the need of external calibration relying only on the physics of diffuse optics. Therefore, it is suited to provide absolute thresholds for guiding interventions. Finally, it has the possibility to investigate the tissue in depth even using a single injection/collection fiber^29^ since the photon travelling time encodes the mean visited depth^30^, that is, longer lived photons have visited tissue regions farther from the injection/detection points^31^. Hence, for clinical translation, it would be valuable to explore the TD techniques to monitor the thermal treatment of biological tissue.

In this work, we report the use of time-domain diffuse optical spectroscopy (TD-DOS) over a broad range of wavelengths (650–1100 nm) to monitor changes in the optical properties of biological tissues with RF-based thermal treatment. We utilize the broadband optical properties to understand the changes in different tissue constituents during treatment and to provide insights into structural alterations. The instrument used in this study is relatively compact and optical fiber-based, suitable for real time monitoring in a clinical setting. Considering the use of thermal treatment for a wide range of biological tissues, we studied the absorption and scattering properties of ex-vivo bovine liver, heart, muscle and brain for critical treatment and overtreatment using a clinical RFA system.

In the following, we present the key results of this study, the first of which is a comparison between optical property spectra of the native (untreated) and completely ablated tissue for all the above-mentioned tissue types for both the critical and over treatment cases. Then, we present the temporal evolution of the whole spectrum and real-time tracking of specific wavelengths during the RFA procedure. We also present the changes to the spectral features when the tissue recovers back to room temperature. This is followed by a discussion on the possible origin of the key observed features and a quick comparison of the results of the current study with those obtained from literature. The final section describes the instrumentation, materials and methods used in this study.

## Results

**Figure 1:**
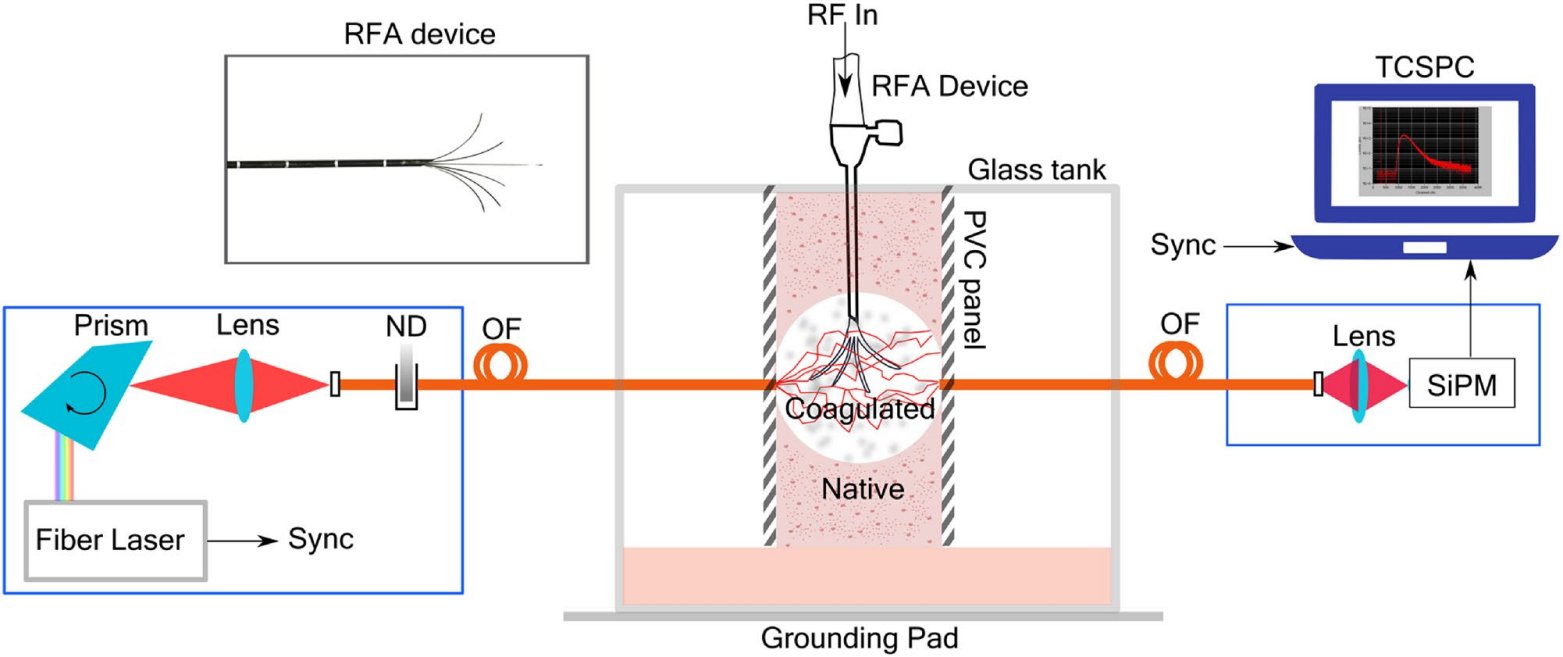
Time-resolved diffuse optical spectroscopy during Radiofrequency ablation (RFA). The measurement of optical properties was performed in transmittance geometry. Light from a broadband (450–1750 nm) fiber laser is wavelength tuned using a Pellin Broca prism and coupled into an optical fiber. A set of circular neutral density attenuators (ND) was used to adjust the incoming laser power. The tissue sample used for thermal treatment is held between two black PVC panels in a glass tank. The RFA device is introduced to the center of the sample and the grounding pad to the aluminum plate at the bottom of the tank. A layer of chicken tissue is placed on top of the grounding pad to increase the surface area which facilitates a uniform growth of thermal lesion in the tissue through the tissue. Alternating current is applied between the RFA device and the grounding pad for heating of the tissue. The detection fiber on the other side of the sample collects the diffusively transmitted light and focuses it into a Silicon Photomultiplier (SiPM) which is then analyzed using a PC based time-correlated single-photon counting (TCSPC) card.

The setup, consisting of the RF-based ablation system and the time-resolved instrument for measuring the optical property spectra, is shown in Fig. 1. The RFA needle device and the fibers are aligned such that optical measurements take place at the center of the ablation zone. The influence of the RFA needle on the retrieved optical properties was studied by comparing the spectra of the tissue with and without the needle and it was found to have negligible effects on the retrieved changes in optical properties (see Supplementary Fig. 1).

To study the effects of critical thermal treatment (temperatures between 50 to 100 °C) and overtreatment (above 100 °C), which results in charring of tissue and is reflected in its optical properties, we have chosen to set the ablation temperature to 70 °C for critical treatment and 105 °C for overtreatment^32^. Once the target temperature was reached at the needle’s surface, the thermal treatment was administered at this temperature for a span of 10 min to allow heat conduction to the region of interest and complete coagulation of the tissue^32^. The wavelength range of choice for a broadband measurement was 650–1100 nm with 10 nm spectral resolution. The acquisition time per spectrum was nearly 1 min. The measurement of the broadband optical spectra for all the tissue types was repeated three times on different samples and the results were found to be in good agreement with each other (average deviation of under 5%).

### Broadband optical properties of the native and thermal treated tissues

**Figure 2:**
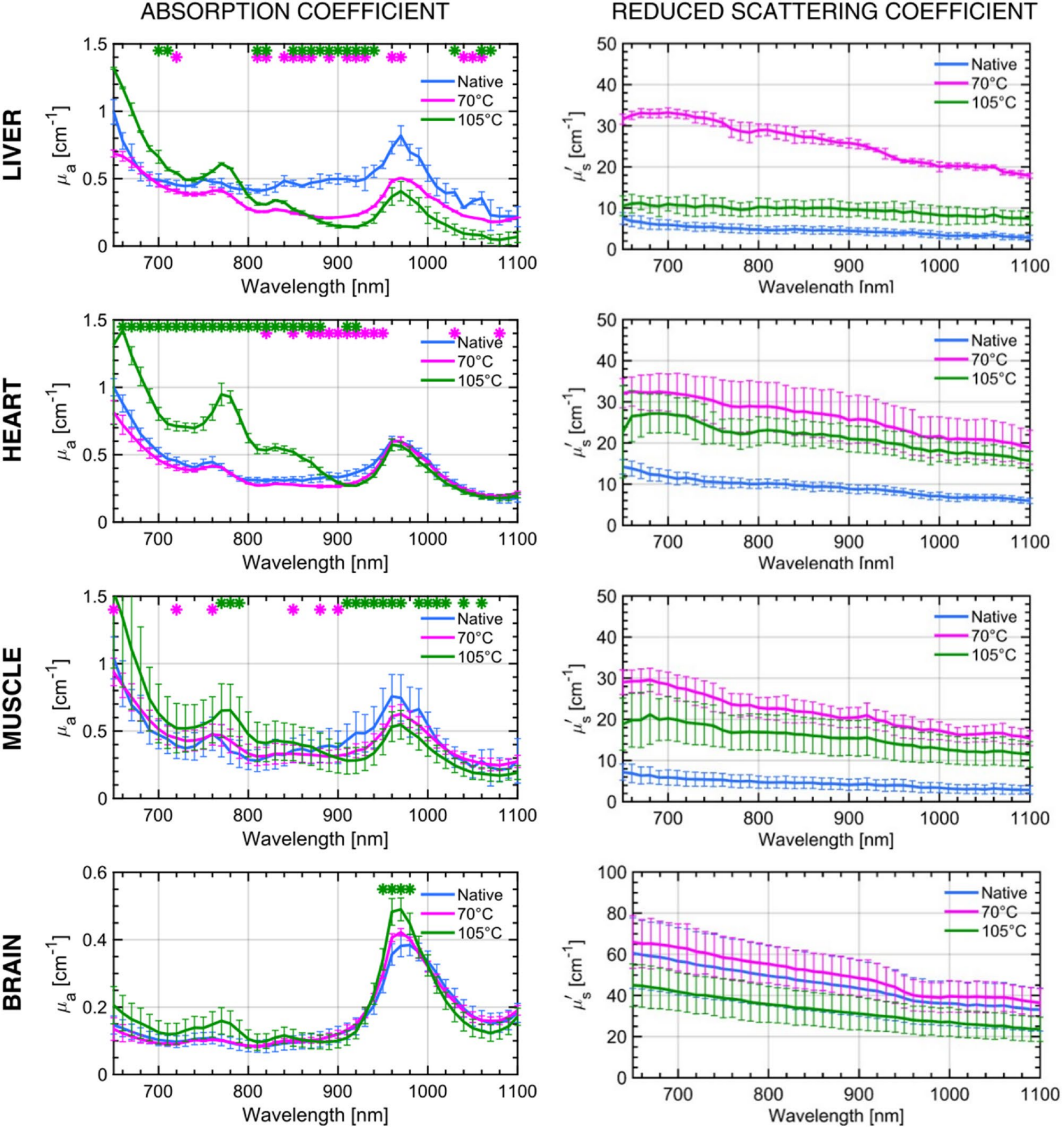
Optical properties of native and treated tissue. Broadband absorption ($\mu_{a}$) and reduced scattering coefficient ($\mu_{s} ^{\prime}$) spectra of native (blue) and tissues treated at two temperatures 70 °C and 105 °C (magenta and green respectively). The tissues are bovine (**a**) liver, (**b**) heart, (**c**) muscle and (**d**) brain. The spectra represent mean values of measurements performed on three different samples for each tissue type and the error bars show the standard deviation over the three measurements. The stars on top of each figure represent the statistically significant (p < 0.05) difference between the treated samples and the native ones at each wavelength.

Figure 2 summarizes the key differences in the broadband optical properties of the native and completely ablated tissue of the four tissue types at the two treatment temperatures (70 °C and 105 °C). The spectra represent the mean values of the absorption ($\mu_{a}$) and reduced scattering coefficient ($\mu_{s} ^{\prime}$) obtained from three different samples, while the error bars represent the standard deviation over the measurements. The stars on top of each figure represent a statistically significant difference calculated at every wavelength between the two treated sample sets and the native tissues. A paired t-test was applied assuming p < 0.05 for statistical difference.

Irrespective of the type of tissue under investigation, two consistent spectral features were observed in the absorption spectra of the native, untreated tissue that are a minor absorption peak at 760 nm and a broader absorption peak around 980 nm. These two peaks have been well studied in literature and correspond to the absorption from deoxy-hemoglobin (Hb) and water, respectively^33,34^. With thermal treatment and attainment of the target temperature, we observe, in general, a complex behavior on the absorption spectrum of the treated state as compared to the native state, with a reduction in the region > 800–900 nm, and an increase in the region < 800–900 nm. Conversely, the scattering coefficient is always increased by a factor of ~ 50–400%, with the only exception of brain treated at 105 °C where scattering reduction is observed, probably ascribed to initial charring, as discussed in the following part. The key variations in the absorption spectra can be assigned to two wavelength windows, the region from 650 to 900 nm and that from 900 to 1100 nm. In the former window, treatment at either of the two temperatures leads to the formation of a new peak at 830 nm. Also, the Hb peak (760 nm) seems to experience a subtle spectral redshift which increases for the higher treatment temperature. In the wavelength region of 900–1100 nm, key variations are observed around the water peak in the form of a drastic reduction in absolute value and a subtle blueshift of the peak. Also, the peak tends to become narrower with thermal treatment. One key difference between the critical treatment and the over-treatment is that in the 600–900 nm region, the absorption seems to experience a further increase in absolute value on over-treatment (105 °C), most of the time even overshooting the native tissue's absorption value.

On the other hand, the reduced scattering coefficient ($\mu_{s} ^{\prime}$) is devoid of any spectral features over the entire wavelength range and follows the expected power-law dependency on wavelength given by an empirical extrapolation of Mie’s law^35^.

$$ \mu_{s}^{^{\prime}} \left( \lambda \right) = a\left( {\frac{\lambda }{{\lambda_{0} }}} \right)^{ - b} . $$

In this case, $\lambda_{0}$ = 650 nm, and the *a* and *b* parameters retrieved from this equation are related to the density and size of the scatterers in the medium, respectively. The values of *a* and *b* are tabulated for the native and coagulated cases at both temperatures, for all the four tissue types in Table 1. In general, we observe a substantial increase in the “*a*” parameter (equivalent density of scattering centers) at both temperatures, and a slight decrease of the “*b”* parameter (effective size) for the overtreatment (105 °C).

**Table 1:** Quantification of the selected features observed in the different tissue types with RF-based thermal treatment at the two temperatures.

Tissue type		Liver			Heart			Muscle			Brain		
Temperature (°C)		23	70	105	23	70	105	23	70	105	23	70	105
Redshift of 760 nm peak (nm)		–	4	15	–	4	13	–	4	18	–	3	14
Blueshift of 980 nm peak (nm)		–	4	5	–	3	3	–	4	5	–	6	9
Scatter parameters	*a* (cm^−1^)	5.6	38.2	12.4	6.9	32.4	21.8	12.5	39.5	33.5	49.8	55.8	47.9
	*b*	1.4	1.5	0.7	1.2	1.4	1.2	1.3	1.4	1.3	1.4	1.3	1.3

The scattering parameters *a* and *b* are related to the density and size of the scattering components in the medium. From the absorption spectra, the relative redshift in the Hb peak and blue shift of the water peak at 980 nm are quantified using a cubic spline interpolation of the measured data.

In the interest of brevity, the figures in the rest of the article mainly deal with the results from the bovine myocardium (heart) tissue, while the figures for the other tissue types can be found in the Supplementary Information.

### Evolution of the broadband optical property spectra during thermal treatment

**Figure 3:**
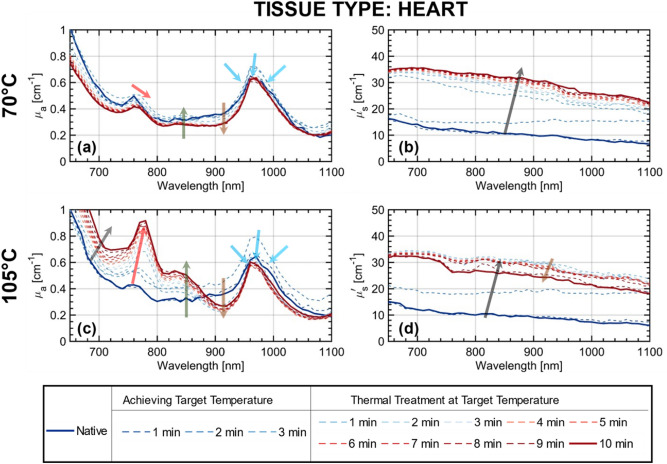
Evolution of the optical property spectra with RF treatment of ex-vivo bovine heart tissue—Broadband absorption *μ*_*a*_ (**a**) and (**c**) and reduced scattering coefficient *μʹ*_*s*_ (**b**) and (**d**) spectra during the RF-based thermal treatment at the two temperatures, 70 °C and 105 °C are shown. The acquisition time per spectrum is 1 min. The RF instrument takes a time of less than 3 min to achieve the target temperatures. The temperature is then maintained for a span of 10 min. Hence in total, a set of 13 spectra are acquired during the RF-based thermal treatment.

Figure 3 shows the temporal evolution of the absorption and reduced scattering coefficient spectra of ex-vivo bovine heart tissue in real-time during the ablation procedure at two temperatures 70 °C and 105 °C, over a period of 13 min. The real-time measurement of the optical properties during the RF treatment, allows us to closely follow the evolution of the spectra as the tissue gets thermally treated. In both cases, slightly less than three minutes are needed to reach the necessary target temperature. Then the treatment is administered at this temperature for a span of 10 min. Here, we observe features in the evolution of the absorption spectra for both treatment temperatures (Fig. 3a,c) with different magnitudes: (i) a redshift of the deoxy-hemoglobin (Hb) absorption peak (760 nm) with increased treatment time; (ii) a subtle blueshift of the water absorption peak (980 nm) along with the reduction of the peak width; (iii) a reduction of the absorption coefficient at 980 nm at both treatment temperatures; (iv) the occurrence of the new peak around 840 nm, though it is more evident in the case of treatment at 105 °C; (v) a substantial dip in the absorption around 910 nm forming a ‘valley’ of a low absorbing region; (vi) an increase in the magnitude of the absorption in the wavelength range 650–850 nm for treatment at 105 °C with increased treatment time.

**Figure 4:**
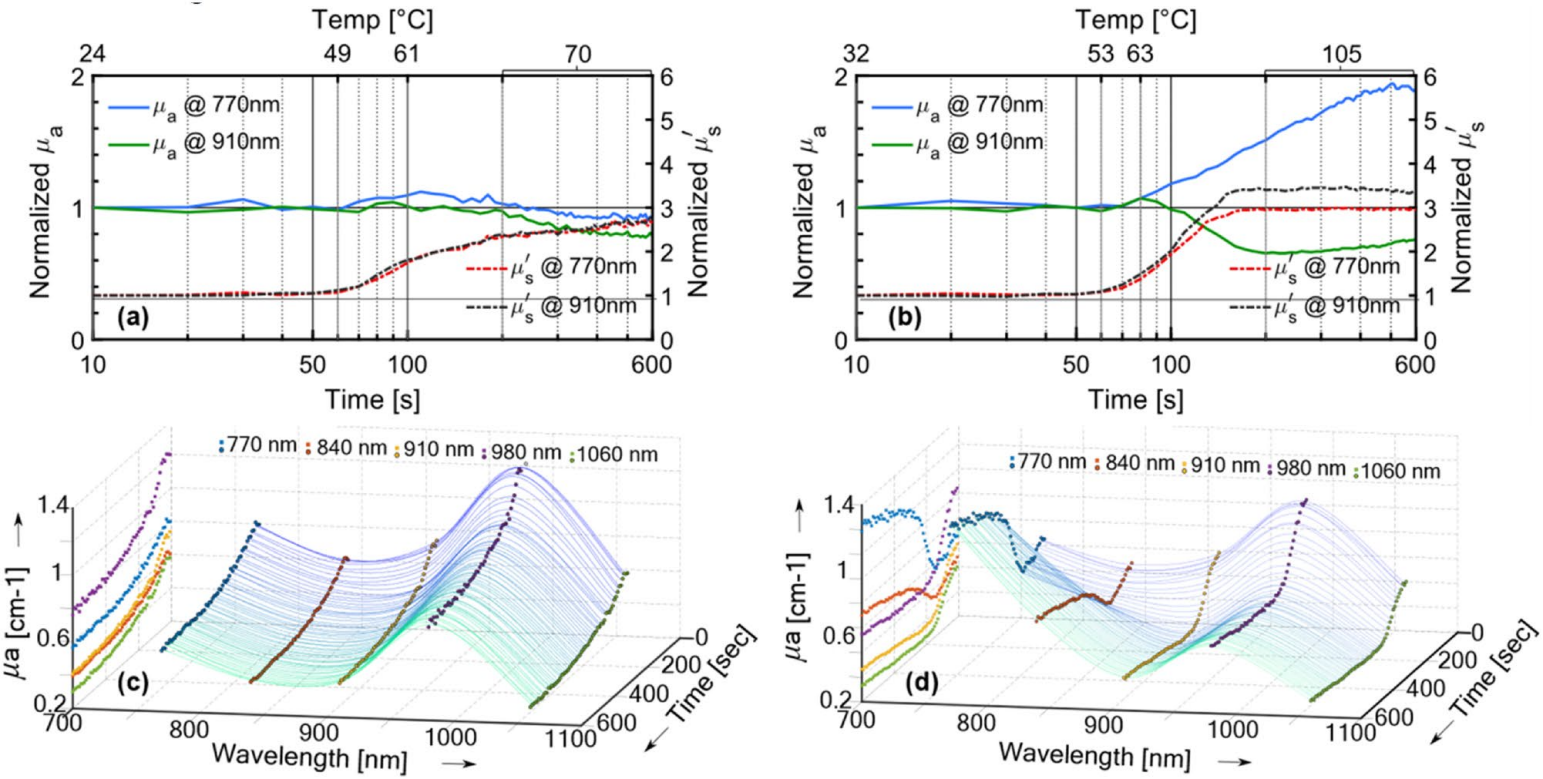
Time evolution of the optical properties at the five selected wavelengths with RF treatment in ex-vivo bovine heart tissue—(**a**,**b**) temporal evolution of absorption and reduced scattering coefficients (*μ*_*a*_ and *μʹ*_*s*_) at two wavelengths (770 and 910 nm) at treatment temperatures of 70 °C and 105 °C respectively. (**c**,**d**) Temporal evolution of *μ*_*a*_ over all the five wavelengths (770, 840, 910, 980 and 1060 nm) for the two treatment temperatures 70 °C and 105 °C respectively. To provide the pseudo-spectral impression, interpolation is used with the five-wavelength information. The time vs. the absorption coefficient for the five wavelengths is projected on 2D plot on the left.

The reduced scattering spectra, in both cases of 70 °C and 105 °C (Fig. 3b,d), mainly exhibit a visible increase in their magnitudes with the RF treatment. For both treatment temperatures, a drastic increase in the reduced scattering is observed over the time it takes for the tissue to reach the target temperature. When treated at 70 °C, the reduced scattering spectrum reaches a maximum value within the first 3 min, i.e. the time required to achieve the target temperature, and hardly experiences any cy.nges beyond that. Heating at 105 °C leads to a similar behavior for the first three minutes, with an increase in the reduced scattering. However, in contrast to the 70 °C case, beyond this time point, there is a gradual decrease in value over the entire spectral range. In both cases, the spectral shape of the reduced scattering spectrum at two minutes is different from the expected power-law dependency. This could be attributed to the rapid transition of tissue structure during the one minute needed for the acquisition of the whole spectrum. In general, the rapid change of properties during the acquisition can alter the shape of both the recovered absorption and scattering spectra. Using the dynamic data presented later on in Fig. 4 we have estimated the temporal derivative of optical properties. In the worst case, the relative change in the spectrum during the 1 min acquisition time is expected to be 20% on absorption and more than 100 more than 100% on reduced scattering. Surely, this creates a deformation of both spectra, that motivated us to provide a finer temporal sampling, as described in the following. Yet, this is the worst case. Out of the critical transition between 1 and 2 min, the distortion is much lower. Since the distortion is smooth over the spectral range the key peaks and spectral features are still clearly visible even in the critical range. Yet, changes in slope are expected as can be observed for the scattering. Given these numbers, the apparent reduction of slope between 1 and 2 min is not real, and in effect the initial spectrum slope is regained after the quick transition. Due to these caveats, a spectral sampling at a much faster acquisition time was pursued, as presented in the following section.

While the relative magnitude of the variations with thermal treatment differs, the key alterations in the broadband optical spectra noted above are also observed (see Supplementary Figs. 2, 3 and 4) in the other three tissue types (liver, muscle and brain). In particular, the evolution of the optical spectra of muscle tissue is in good agreement with that of the myocardium tissue. In the case of liver tissue, the results are consistent for treatment at 70 °C, but when treated at 105 °C the further decrease in the reduced scattering spectra is substantially larger. Finally, in the brain tissue, contrary to the other three tissue types, we observe an increase in the absorption coefficient and reduction in the reduced scattering coefficient upon treatment. The peak shifting, peak formation at 840 nm and peak narrowing effects, are still evident and consistent with the other tissue types.

### Time evolution of optical properties

The continuous time evolution of the optical properties at a few specific wavelengths during treatment is plotted in Fig. 4 for the two treatment temperatures. The variations in the broadband absorption spectra with thermal treatment (Fig. 3) suggest that the time evolution of the optical properties is strongly dependent on the selected wavelength. To closely observe the above-mentioned effects, the time course was measured for five wavelengths, where noteworthy behavior had been observed as in Fig. 3. These chosen wavelengths are 770 nm (position of the redshift), 840 nm (emergence of the new peak), 910 nm (emergence of the new valley), 980 nm (position of the blue shift) and 1060 nm (emergence of the valley). For each of these wavelengths, the measurements were acquired once every 10 s for a span of 600 s. The 600 s of measurement also includes the initial three minutes required to reach the target temperature for thermal treatment.

Figure 4a,b compare the time evolution of both the absorption coefficient and reduced scattering coefficient at 770 and 910 nm, for treatment temperatures of 70 °C and 105 °C, respectively. The time axis is plotted in log scale to better appreciate the trends in the non-linear heating. The temperature (average of the four electrodes of the RFA device) is marked at specific points in time at the top of the figures. The temperature follows a linear increase until the target goal is reached and then remains constant up to the end. The dissimilarity in the trends of the absorption and reduced scattering coefficients implies that there is no crosstalk or influence of one optical property on the other. The optical properties are normalized with respect to their initial values for a better understanding of their evolution. When treated at 70 °C (Fig. 4a): (i) the absorption coefficient both at 770 nm and at 910 nm remains invariant during the time it takes to reach the target temperature. Beyond this point, the absorption coefficient at both wavelengths starts to decrease steadily (larger magnitude at 910 nm). (ii) The reduced scattering remains constant as that of the native untreated tissue, until 50 s into the treatment when the tissue temperature is just about 50 °C. Then a sudden drastic increase is observed until the target temperature (70 °C) is reached, doubling in value with respect to the native state at around the 200 s mark. Beyond this point (and when the treatment is administered at the constant temperature of 70 °C) the reduced scattering undergoes a slow increase until the end of the treatment time of 600 s. The temporal evolution of the normalized *μʹ*_*s*_ at both wavelengths is almost identical. When treated at 105 °C (Fig. 4b): (i) The absorption values are again constant as in the previous case for the first 1 min. However, beyond this point the absorption at 770 nm experiences an increase in amplitude to almost double the native value at the end of the 10-min treatment. On the other hand, the value at 910 nm decreases gradually until the 200 s mark (~ 105 °C) beyond which it stabilizes with a little rise at the very end of the treatment time. (ii) The reduced scattering experiences a sigmoid like shape. The normalized *μʹ*_*s*_ is invariant for the first 60 s, followed by a period of fast and drastic increase to 3 times of the native value. Beyond 180 s or 3 min, the reduced scattering remains unchanged for the rest of the treatment time.

Figure 4c,d plot the absorption coefficients sampled at the five wavelengths, with the z-axis showing the changes in time, for treatment temperatures of 70 °C and 105 °C, respectively. The projections on the time vs optical absorption plane (left plane for each graph) compares the time courses of the absorption coefficient for the five wavelengths. Since the chosen wavelengths were equidistant for the most part, such a visualization describes the temporal evolution, as well as the wavelength dependence of the optical properties albeit at a lower spectral resolution. The observed features in Fig. 4a,b for 70 °C and 105 °C treatment hold true here as well, with the absorption coefficient at 840 nm having a similar behavior like that at 770 nm, and the absorption coefficient at 980 and 1060 nm following the behavior at 910 nm.

In general, similar trends in broadband spectra with treatment are observed for liver and muscle tissue (see Supplementary Fig. 5). In the case of brain tissue, variations in the behavior are observed at 70 °C that are: (i) the relative change between the native and treated tissue’s absorption coefficient is negligible; (ii) the reduced scattering experiences only a 20% increase in magnitude over the treatment time. With treatment at 105 °C, the brain tissue response shows a deviation from the sigmoidal evolution of optical properties seen in the other tissue types. Rather, the absorption at 910 nm, and reduced scattering at both the wavelengths 910 and 770 nm monotonically decrease with time with a minor peak at the 100 s mark. The absorption at 770 nm, on the other hand, increases monotonically. The relative magnitude of change in both the optical properties is under 10% over the entire measurement duration.

### Variation of optical properties on passive cooling of the ablated tissue

**Figure 5:**
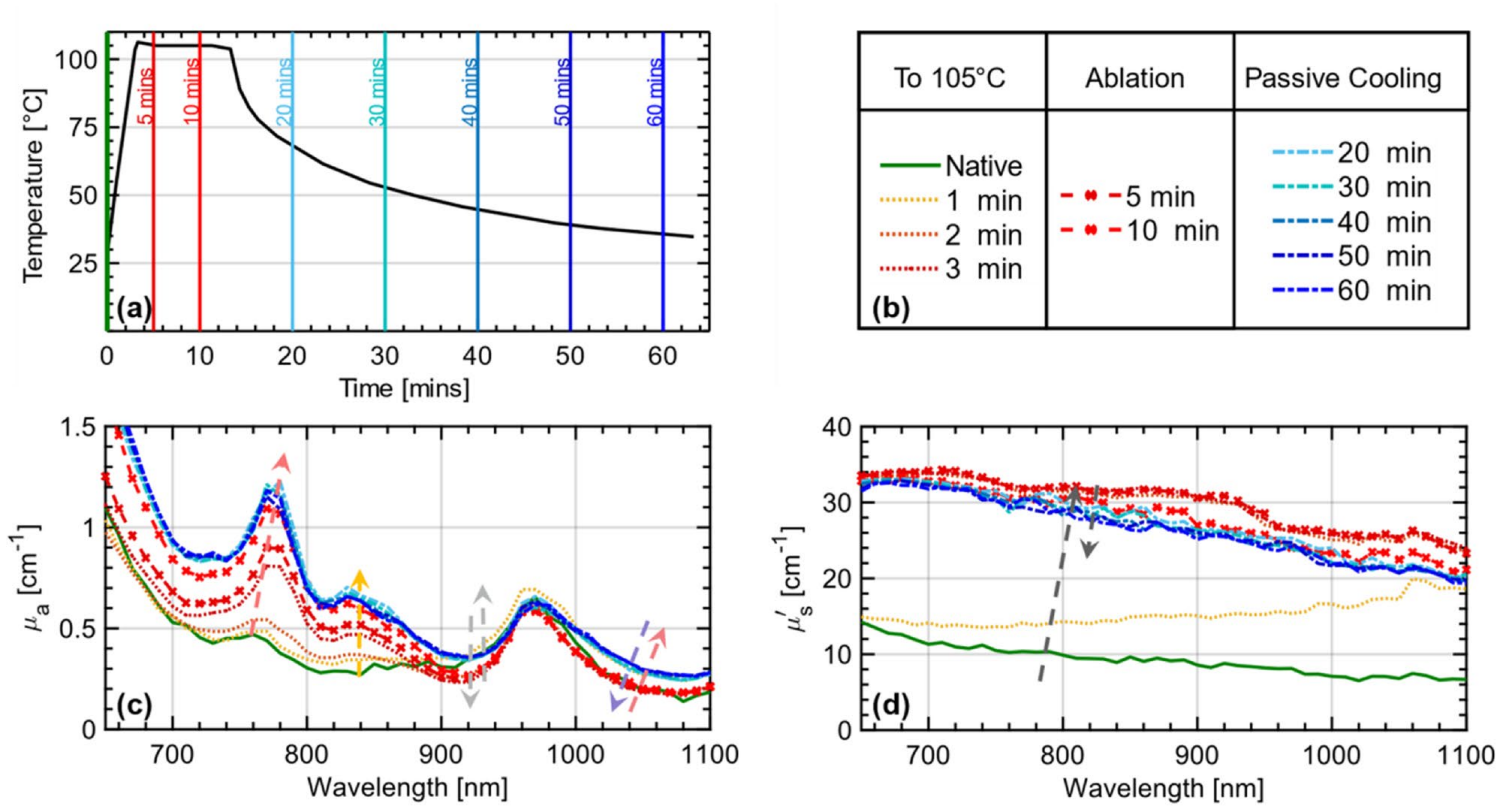
Reversibility of optical properties after cooling down of the ablated bovine heart tissue: (**a**) Average electrode temperature measured inside the heated volume, during the heating-up, ten-minute ablation at 105 °C, and cooling down of the bovine heart tissue. (**c**) Absorption spectra and (**d**) reduced scattering coefficient spectra at the different time stamps as shown in the legend (top right).

As we have observed in the previous sections, thermal treatment alters the amount of absorption and scattering and spectrally shifts some of the absorption peaks. To test if these changes are reversible, optical properties were determined during the cool-down of ablated tissue. The treatment procedure remains the same as described earlier, with all tissue types being treated for 10 min, at both 105 °C and 70 °C. Directly following the ablation, the tissue was allowed to cool down passively to room temperature (~ 23 °C). During this period, the optical properties were acquired for the spectral range 650–1100 nm at 10 nm step size. Figure 5 consolidates the results for bovine heart tissue during and following the ablation at 105 °C.

The changes in optical properties with cooling-down are as follows. (i) The hemoglobin absorption peak at 760 nm, which underwent a redshift of 15 nm (refer Table 1) did not show any additional shift during cooling-down. However, a small increase in the absorption coefficient was observed. (ii) The new peak observed at 840 nm during heating, showed a small increase in magnitude during cooling down. (iii) The valleys formed at 910 and 1060 nm during heating, partially reversed with cooling. (iv) The water absorption peak at 980 nm experienced a minor red shifting of the peak and a partial recovery of the peak width and magnitude on cooling. Passive cooling has a negligible effect on the optical property spectra and the observed signatures of the tissue treated at 70 °C (results not shown here).

## Discussion

Systematic variations in the optical properties of tissue undergoing thermal treatment could be of great interest in closely monitoring the dosage and spatial extent of the treatment in real-time. In this work, we uncover certain unique changes in tissue absorption and reduced scattering spectra upon RF-based thermal treatment. These optical properties have been measured using Time-Domain (TD) diffuse optics, an approach that improves the accuracy and reliability of the results in comparison with previously reported results based on Continuous Wave (CW) techniques^18–24^.

Key variations were observed at five narrow spectral bands in the absorption coefficient, while the reduced scattering coefficient mainly experiences a global wavelength-independent increase and/or decrease in absolute value. Partial reversibility of certain variations was observed on the passive cooling down of the tissue to room temperature after thermal treatment. A quantitative description of some of the key variations observed in the optical properties for all the tissue types is summarized in Table 1. Since the spectral measurement was performed with a step size of 10 nm the resultant shifts described in the table were obtained by interpolating the measured spectra with a cubic spline interpolation^36^.

In the following, we attempt to understand the possible origins of the different variations observed in this study. Molecular spectroscopy suggests that a biochemical change occurring in the tissue with the thermal treatment manifests itself as a peak shift or peak formation in the absorption spectrum while a variation in the magnitude of the optical property is indicative of a physical or structural change. Starting with the absorption spectrum, we observe a general decrease with thermal treatment. This result is in agreement with previous studies^14,18,23,24,37^. Some of these studies suggest the destruction of different absorbing chromophores like hemoglobin and cytochrome oxidase with heating as a possible reason for the decrease in the absolute value. Additionally, we also observed variations in three regions in the absorption spectra: (i) the hemoglobin peak around 760 nm; (ii) the peak around 840 nm; and (iii) the water peak around 970 nm. We consider each of these regions individually and discuss these variations in further detail.

Table 1 shows that redshifts of roughly 5 nm and 15 nm of the hemoglobin peak are observed in all the tissue types when treated at 70 °C and 105 °C, respectively. We explain this behavior by invoking the explanation forwarded by Black et al.^38,39^ in their studies on the chemical and structural changes of whole blood undergoing photocoagulation. In these studies, measurements of reflectance and transmittance profiles on blood samples undergoing laser photocoagulation showed a similar hemoglobin redshift. This behavior is explained using vibrational and molecular spectroscopy. At elevated temperatures, the vibrationally excited levels of the electronic ground state of hemoglobin are populated. This allows for transitions with lower energies (or longer wavelength) giving rise to the observed redshift in the absorption spectrum. This appears to be a plausible explanation for our observations of the hemoglobin redshift^39^. The increase in the magnitude of the shift when treated at a higher temperature follows as a direct consequence.

**Figure 6:**
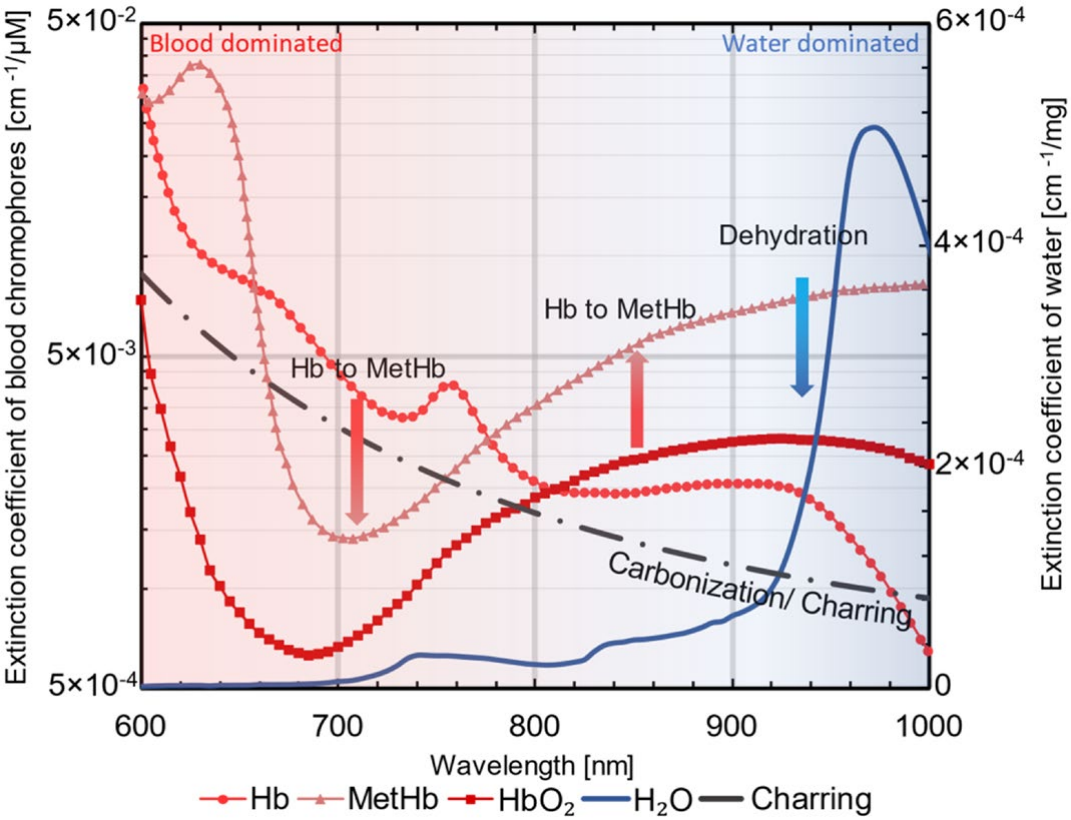
Extinction spectra of key absorbers in biological tissue undergoing thermal treatment. Blood chromophores (Hb, HbO_2_ and MetHb) are plotted against the logarithmic y axis on the left. While the spectrum of water is plotted against the y-axis to the right. The dotted line decaying exponentially with wavelength represents the influence of charring on the absorption. The absorptivity on either side of the 800–900 nm wavelength region decreases with treatment. While the absorptivity in the region of 800–900 nm increases with thermal treatment as a consequence of the conversion of Hb and HbO_2_ into MetHb. (Figure generated using data from omlc.org/spectra).

Another interesting observed feature is the increase of absorption over the wavelength range of 650–800 nm with over-treatment. To explain this, we invoke some other thermally induced biochemical changes that are known to occur in the blood chromophores at elevated temperatures. First, at high temperatures (above 75 °C), blood chromophores have been shown to undergo a transition into a modified species known as methemoglobin (metHb)^39^, a form of hemoglobin in which the iron exists in the Fe(III) oxidation state, incapable of exchanging molecular oxygen in tissue. This conversion is further confirmed in vivo by studies performed on subjects with port-wine stain and telangiectasia^40^. MetHb has a greater absorptivity over the other two blood chromophores (Hb and HbO_2_) in the wavelength region of 600–1100 nm. The increase in absorptivity as a consequence of this conversion could explain the further increase in the absorption coefficient that we observe for all the tissue types when treated at a temperature of 105 °C. Further confirmation of this hypothesis is seen in Fig. 4b, where the absorption at 770 nm shows an increase when the treated region attains an average temperature of 82 °C. Secondly, the overtreatment scenario (having a target temperature of 105 °C) with ablation for 10 min, usually resulted in the formation of a blackish carbonized layer (charring) in the treated area for all the tissue types. This carbonization, which has been shown to have an exponential dependence with wavelength (Fig. 6) on the absorption spectrum^41^, displays a greater increase in the visible than in the NIR wavelength region. Finally, the redshift of the absorption bands of hemoglobin (as discussed earlier) means that the characteristic high absorption bands (in the visible wavelength region) are now shifted to longer wavelengths, further increasing the value of absorption in this region. Thus, the substantially high increase in absorption observed in the region between 650 and 800 nm can be attributed to a combination of these three factors.

The formation of the peak at 840 nm, a feature consistent among all the tissue types and more evident for the overtreatment case, has been reported here for the first time. The origin of the peak remains unclear at this point. The only observable peak at 840 nm is one from the water absorption spectrum^42^. However, even at high temperatures, this peak is of negligible magnitude (relative to the other water peak at 980 nm) and cannot explain the relatively high value of absorption observed in this study. One other hypothesis for the occurrence of this peak is the above-mentioned conversion of Hb to metHb at an elevated temperature. The spectral features of these two chromophores suggest that this conversion, when considered in isolation, implies a net reduction in the absorption below 780 nm (since metHb has a lower absorption compared to Hb as shown in Fig. 6). The opposite, however, is true in the 780–900 nm range where metHb dominates in absorption over the other two blood chromophores. Finally, beyond 900 nm water becomes the key absorber and as a consequence of dehydration of the tissue with over-treatment, the absorption in this region experiences a drastic reduction. Considering these variations together, we can offer a tentative explanation of the occurrence of the 840 nm peak with heating, a feature in this region of the spectrum which is absent in the native absorption spectrum. While this hypothesis needs further confirmation, monitoring the generation and evolution of this peak could give useful insight on the conversion of blood chromophores into metHb.

A blue shift and narrowing of the peak at 970 nm are consistently observed in all the tissues under study. Table 1 suggests that this shift is roughly 5 nm when the tissue is treated at 105 °C, and 4 nm when treated at 70 °C. Since the absorption around 970 nm is dominated by water, these effects could be due to changes in the water with temperature. Some clues can be found in multiple studies^42,43^ where a similar blue shift in the absorption peak and band narrowing with temperature is reported. Chung et al.^43^ attribute both these features to the weakening of the hydrogen bonding of water molecules with a rise in temperature. It was also mentioned that depending on whether the bonding is between two water molecules or between water and the other macromolecules present in the tissue, the directionality of the shift in this absorption peak could change. The partial retrieval in both the peak width and peak position on cooling, shown in Fig. 5, becomes a very interesting feature in this light. This recovery could then be explained as a consequence of the chemical transformation of the remnant water in the tissue to its native state on cooling. However, this could also be a result of the water from the surrounding untreated region of the tissue, gradually occupying the relatively dry treated area during the passive cooling. In any case, further studies with specific attention to the dynamics of the water content in the region of treatment of the tissue are necessary to better understand these effects. The reduction of absorption around the water peak is a rather straightforward consequence of the net reduction of the water content in the tissue (dehydration).

The reduced scattering spectra experience a drastic increase in the magnitude from the native to the ablated or coagulated stage, and this behavior is consistent for all tissue types except for the brain (Fig. 2), and at both temperatures (Fig. 3b,d). The scattering parameters *a* and *b* (from the Mie power-law), which are a measure of the density and size of the scattering centers respectively are presented in Table 1. A consistent three- to four-fold increase in the scatter density is observed in all the tissue types except for the brain, for both temperatures. The subsequent decrease in scattering means that the *a* parameter at 105 °C is sometimes lower than that obtained by thermal treatment at 70 °C. This drastic increase in scattering with thermal treatment has been observed earlier in many ex-vivo and in-vivo studies^18–21^. The thermal breakdown could lead to a change in the tertiary protein structure, which results in an increased scattering density^18^. Regarding the size of the scattering centers, a study performed on rat liver, using a 1064 nm laser to induce thermotherapy in-vivo, showed a decrease in the *b* parameter with thermal coagulation, which is in agreement with our findings for bovine liver tissue at 105 °C^44^. Further, the reported values for the *b* parameter for ex-vivo bovine liver (1.2 for untreated, 0.9 for thermally treated tissue), are similar to values obtained in this study (1.4 for untreated and 0.7 for treated tissue). The decrease in the reduced scattering spectrum on overtreatment as compared to normal treatment at 70 °C, is simultaneous to the further increase in absorption. We think that this is an outcome of the carbonization of the tissue.

The measurements performed during passive cooling of the tissues display partial recovery of some features mainly in the water absorption band. This primarily pertains to the partial re-widening of the peak width, which is indicative of partial recovery to the native state, of biochemical interactions between water and macromolecules. The former in combination with the diffusion of water from the untreated regions into the treated area could also contribute to this observation. The valley observed around 910 nm seems to increase during cooling, believed to be due to the same effects. The additional increase in the absorption at 840 nm with cooling, could further validate the assumption about the conversion of blood chromophores into metHb since carbonization is no longer expected during the cooling period. And for temperatures above 75 °C, metHb can still be formed, thus during the cooling process the peak is able to increase at those temperatures. The same is believed to be true about the 760 nm peak, also showing an increase during cooling. A minor reduction in the reduced scattering over all wavelengths with cooling was observed compared to the ablated state, the reason for this remains unclear.

Results from the liver and muscle tissues are, in general, consistent with those obtained from the heart (myocardium) tissue. Thus, all the above-mentioned discussion is well-suited to both these tissue types. The lower reduced scattering coefficient with overtreated tissue compared to 70 °C treated tissue, is more evident in these two tissue types and is possibly a consequence of their tissue microstructure evidenced by histological studies^15,18,21,22^. Brain tissue, on the other hand, shows exceptional features in some cases. Contrary to the other three tissues, the absorption spectrum generally increases upon treatment. Similarly, unlike the other tissues, we observe a decrease in reduced scattering with treatment. Apart from these two features, all the other observations are in good harmony with the other tissue types. Though non-invasive optical measurements are routinely performed on the brain, as in functional brain monitoring, the thermochemistry of this tissue must be understood to better interpret these results. Also, the optical properties of the brain are substantially different with respect to liver, muscle and heart, with a much higher scattering coefficient and lower absorption, reflecting the diverse tissue composition and structure.

One of the limitations of this study is the usage of non-diseased ex vivo tissue, which demands certain caution. Studies performed on the optical properties in vivo and ex vivo on mouse ear models found that in the ex vivo case, the optical properties decrease over time due to loss of blood^45^. All the observations in this study were performed as a comparison between the thermally treated tissue and the native tissue, thus the factors such as the influence of the radiofrequency needle on the retrieved optical properties can be neglected. Simultaneous histological assessment and use of thermal damage models^46^ of the tissue could help in better understanding the extent of thermal damage in the tissue and its correlation with the recovered optical properties. Microbubble formation in the tissue during the RFA procedure might have also contributed to the increase in scattering during the procedure and a decrease in scattering while cooling down. Future studies are aimed to confirm the findings on live animal models, both healthy and diseased, before conducting studies intraoperatively in clinics. Other future directions could be using spatially resolved optical imaging methods, such as photoacoustics^47,48^ to assist in differentiating ablated and non-ablated regions or up-scaling the point spectral measurement to Diffuse Optical Spectroscopy Imaging using multiple fibers to obtain ablation maps.

## Conclusions

In conclusion, real-time and accurate monitoring of the optical properties of tissue undergoing thermal treatment can be of great interest in deciding the dosage and treatment duration. This work demonstrates certain unique and consistent features in the broadband absorption and reduced scattering coefficient spectra of biological tissue undergoing thermal treatment. The possible physical and biochemical nature of the origins of these variations are also discussed. To our knowledge, this work demonstrates for the first time, the use of broadband time-domain (TD) diffuse optical spectroscopy for this purpose. The ability of the TD technique to continuously monitor the treatment procedure, even if at a few wavelengths, indicates that this technique could provide valuable real-time feedback on the efficiency and efficacy of the thermal treatment. The monitoring procedure could be carried on even after the termination of the thermal treatment to track changes in the optical properties of the tissue post-ablation. This study provides a wealth of information in the form of optical properties which could be used to design reliable markers or metrics that predict the extent of tissue ablation. The key wavelengths identified can be used to develop single wavelength monitoring systems that can be incorporated into the RFA device. Future lines of investigation will be to study the effects and their correlations with the extent of thermal damage and ablation margins, in malignant tissue in vivo.

## Methods

In this section, we present the setup used in this study consisting of a Time-Resolved Diffuse Optical Spectrometer (TR-DOS) system and a clinical Radio Frequency Ablation (RFA) system. Also, tissue preparation, measurement protocols, and data analysis are discussed.

Figure 1 shows a schematic of the setup used in this study. The sample was placed in a glass tank where the heat treatment and optical monitoring could take place simultaneously. Tissue was gently clamped between two black PVC (polyvinyl chloride) plates inside the glass tank. A hole was drilled in each plate wide enough to allow optical fibers to maintain perfect contact with the tissue. The holes in the plates were placed opposite to each other, enabling the optical measurement to take place at the center of the ablation volume. The black PVC plates was chosen to prevent stray light and avoid any interference to the RF treatment procedure. The RFA applicator device was inserted into the tissue orthogonal to the direction of the light transmission in the diffuse optics measurement (see Fig. 1). The RFA device was fixed to a post and the tines\electrodes were extended to enclose a spherical volume 1 cm in diameter. Scale markings provided on the RFA device were used to position the device such that the optical axis passes right through the center of the ablation zone.

### Time-resolved diffuse optical spectrometer

The optical chain of the time-domain diffuse optical spectrometer used in this study is presented below. A broadband pulsed supercontinuum fiber laser (SuperK EXW-12, NKT Photonics, Denmark) operating at a repetition rate of 80 MHz was used as the source. A Pellin Broca prism disperses the white light pulses which are then coupled into a graded-index optical fiber (50-μm core multimode fiber). Wavelength selection is achieved by rotating the prism. The maximum output power at this stage is between 0.5 and 10 mW, depending on the selected wavelength. The nonlinear dispersion of the prism causes a variation in the pulse bandwidth with wavelength (3 nm at 650 nm to 9 nm at 1100 nm). A tunable circular neutral density filter adjusts the power at the sample, avoiding detector saturation. All measurements in this experiment were performed in transmittance. Light diffusely transmitted by the sample is collected by another 1 mm diameter step-index fiber and coupled to a 1.3 × 1.3 mm^2^ area silicon photomultiplier detector (Hamamatsu Photonics KK) with home-made front-end electronics^49^. The signal from this detector is then finally fed into a TCSPC board (SPC-130, Becker & Hickl, Germany), which acquire and store time-resolved data for analysis. The detector has a good detection efficiency (around 30% at 600 nm and 10% around 800 nm), a dark count rate under 50-kilo counts per second, and an afterpulsing probability below 1%. The system as a whole has a good temporal resolution (FWHM < 90 ps over the entire wavelength range). The wavelength selection, source attenuation, and signal acquisition procedures were completely automated to ensure speed and avoid manual errors. The acquisition time of each spectrum (650–1100 nm) was tailored and fixed to exactly 1 min. The system has been extensively characterized following internationally agreed protocols^50^ and was found to be reliable and accurate in its standard operating conditions. A detailed description of the system can be found elsewhere^51,52^.

### Radiofrequency ablation system

For the ablation procedure, we used a clinical RFA system consisting of an RF generator (1500X, Angiodynamics) to generate an alternating current of 460 kHz through the RFA applicator device (Angiodynamics Starburst XL) towards the grounding pad. During the ablation, the power was set to 150 W. The electrodes of the RFA device led the alternating current through the tissue towards the grounding pad which was connected to the conducting aluminum bottom of the tank. The effect of the metal electrodes (tines) in the light propagation path on the optical properties was found to be negligible (see Supplementary Fig. 1).

To spread the electric current coming from the needles across the entire grounding pad, we covered the bottom plate with 2 cm thick chicken tissue, which was purchased from the local supermarket. This is essential because the difference in surface area between the tines and the grounding pad makes the heat to be concentrated around the tines^1^, allowing for local ablation to occur. The temperature of the tines was displayed on the control panel of the RF generator. The temperature measurement allows the RF generator to control the ablation temperature. Tracking of the measured temperatures in time was performed by filming the display, this was used to correlate the spectral changes with temperature when required.

### Sample preparation

Four ex-vivo tissue types were used in the ablation measurements: Veal muscle and bovine brain, heart, and liver. The tissues were taken from a local butcher and moved in a portable refrigerator to the laboratory, contact with water was avoided. Samples for investigation were pre-cut into slices of 1 cm thickness and roughly 7 × 7 cm (matching the dimensions of PVC plates). After cutting they were put into a plastic bag which was then vacuumized and kept in a refrigerator around 1.5–2 °C. The maximum time of tissue between storage and measurement was 5 days. At the start of each measurement, the tines were employed to a diameter just smaller than the distance between the plates.

### Measurement protocol

When the temperature of tissue enters the region between 60 and 100 °C, immediate coagulation of tissue occurs, but when the temperature gets higher than 100–110 °C, the tissue starts to suffer from charring, retarding the ablation procedure^1^. Because charring is aimed to be avoided in clinical settings, we investigated the change in optical properties during the ablation procedure of several tissues with an RFA target temperature of 70 °C (lowest possible setting). At the start of the procedure, the tissue is heated towards the target temperature, when the average of those temperatures is reaching this temperature, that temperature is then maintained, ablating the tissue for a period of time one can select. We chose to maintain an ablation time of 10 min, to investigate how the optical properties would change over time. Measurements were also performed at a temperature of 105 °C to mimic the situation when charring would occur, allowing us to investigate the effect of charring on the optical properties. For each conducted experiment, first a measurement before starting the heating procedure was acquired, resulting in a native spectrum. Then the tissue was heated and ablated for 10 min, during which each minute optical properties were measured for wavelengths of 650–1100 nm in steps of 10 nm. We defined the last spectrum obtained during the 10-min ablation as the ablated spectrum.

### Data processing

For most of the tissue types considered in this study, the native tissue displayed high absorption and low scattering in the wavelength region of interest. This could heavily influence and distort the results from the diffuse approximation (DA) of the radiative transfer equation (RTE), which is the usual analysis method employed for such studies. For high absorption or low scattering, a fitting procedure based on Monte Carlo (MC) simulations outperforms the DA of the RTE^53^, therefore this method was used to extract the optical properties of the samples. A library of MC simulated temporal point spread functions (TPSFs) for transmittance geometry was generated using a CUDA (Compute Unified Device Architecture) accelerated MC code^54^ to cover a range of scattering coefficients at null absorption. Then, the Lambert–Beer exponential term exp(-*μ*_*a*_*vt*) (where *μ*_*a*_ is the absorption coefficient and $\nu$ is the speed of light) is multiplied to this TPSF to account for the absorption. Interpolation and the scaling property of the RTE then allow for a fast search of the correct simulation to extract the optical properties. The analysis considers the broadening of the pulse occurring due to the instrumentation by convolving the simulated TPSFs with the instrumental response function (IRF) before fitting it to the experimental data. The fitting was performed over the region of the temporal curves with counts higher than 80% on the rising edge and 1% on the falling edge. The refractive index of the sample was considered to be 1.41 (corresponding to bovine tissue) and the external media on either side of the sample was considered to be 1.5 (corresponding to the PVC panels). The time taken to extract the properties at 1 wavelength on a standard PC is less than a second. Also, in-house software allows for the ‘online’ analysis of the data-parallel to its acquisition thus making it suitable for real-time usage.

## Supplementary information

Supplementary Information.

## Data Availability

The data that support the plots within this paper and other findings of this study are available from the corresponding author upon reasonable request.
