# Author Correction: Optical signatures of radiofrequency ablation in biological tissues

**DOI:** 10.1038/s41598-021-96881-9

**Published:** 2021-09-01

**Authors:** Pranav Lanka, Kalloor Joseph Francis, Hindrik Kruit, Andrea Farina, Rinaldo Cubeddu, Sanathana Konugolu Venkata Sekar, Srirang Manohar, Antonio Pifferi

**Affiliations:** 1 Department of Physics, Politecnico di Milano, Milan, Italy; 2 Multi‑Modality Medical Imaging Group, University of Twente, Enschede, The Netherlands; 3 Biomedical Photonic Imaging Group Technical Medical Centre, University of Twente, Enschede, The Netherlands; 4 Institute of Photonics and Nanotechnologies, National Research Council, Milan, Italy; 5 Biophotonics@Tyndall, IPIC, Tyndall National Institute, Lee Maltings, Dyke Parade, Cork, Ireland

Correction to: *Scientific Reports* 10.1038/s41598-021-85653-0, published online 22 March 2021

The original version of this Article contained errors in references 3, 4, 25, 36, 41 and 48, which were incorrectly given as:

3. Neurosurgery, L. O.-S. and F. & 1976, undefined. Electrophysiologic principles of radiofrequency lesion making. *karger.com.*

4. Choi, E., Choi, Y., Jang, E., J. K.-T. K. journal of & 2016, undefined. Neural ablation and regeneration in pain practice. *ncbi.nlm.nih.gov.*

25. Venkata Sekar, S. K. *et al.* Broadband time domain diffuse optical reflectance Spectroscopy: A review of systems, methods, and applications. *Appl. Sci. (Switzerland)* https://doi.org/10.3390/app9245465 (2019).

36. de Boor, C. *L-436 A Practical Guide to Splines* (Springer, 1978).

41. Optics, S. J.-A. & 1993, undefined. Role of tissue optics and pulse duration on tissue effects during high-power laser irradiation. osapublishing.org.

48. Francis, K., Biology, S. M.-P. in M. & & 2019, undefined. Photoacoustic imaging in percutaneous radiofrequency ablation: device guidance and ablation visualization. *iopscience.iop.org.*

The correct references are listed below:

3. Organ, L. Electrophysiologic Principles of Radiofrequency Lesion Making. *Stereotactic and Functional Neurosurgery* **39**, 69-76 (1976). https://doi.org/10.1159/000102478

4. Choi, E. *et al.* Neural Ablation and Regeneration in Pain Practice. *The Korean Journal of Pain* **29**, 3-11 (2016). https://doi.org/10.3344/kjp.2016.29.1.3

25. Konugolu Venkata Sekar, S. *et al.* Broadband Time Domain Diffuse Optical Reflectance Spectroscopy: A Review of Systems, Methods, and Applications. *Applied Sciences* **9**, 5465 (2019). https://doi.org/10.3390/app9245465

36. Boor, C. A practical guide to splines. (1978). ISBN 978-0-387-95366-3

41. Jacques, S. Role of tissue optics and pulse duration on tissue effects during high-power laser irradiation. *Applied Optics* **32**, 2447-2454 (1993). https://doi.org/10.1364/AO.32.002447

48. Francis, K.J. & Manohar, S. Photoacoustic imaging in percutaneous radiofrequency ablation: device guidance and ablation visualization. *Physics in Medicine & Biology* **64**, 184001 (2019). https://doi.org/10.1088/1361-6560/ab36a1

The original Article has been corrected.

