# Supplementary material for: Optical signatures of radiofrequency ablation in biological tissues
Source: Sci Rep. 2021 Mar 22;11:6579. doi: 10.1038/s41598-021-85653-0 (PMC7985316; doi:10.1038/s41598-021-85653-0)
Supplement: Supplementary file 1 — Supplementary Information. [file 41598_2021_85653_MOESM1_ESM.docx]

Supplementary Data: Optical signatures of Radiofrequency Ablation in biological tissues

Pranav Lanka^1*^, Kalloor Joseph Francis^2*^, Hindrik Kruit^2*^, Andrea Farina^4^, Rinaldo Cubeddu^1^, Sanathana Konugolu Venkata Sekar^5^, Srirang Manohar^2^ and Antonio Pifferi^1,4^

# **^Difference in optical properties with and without RFA device in the sample.^**


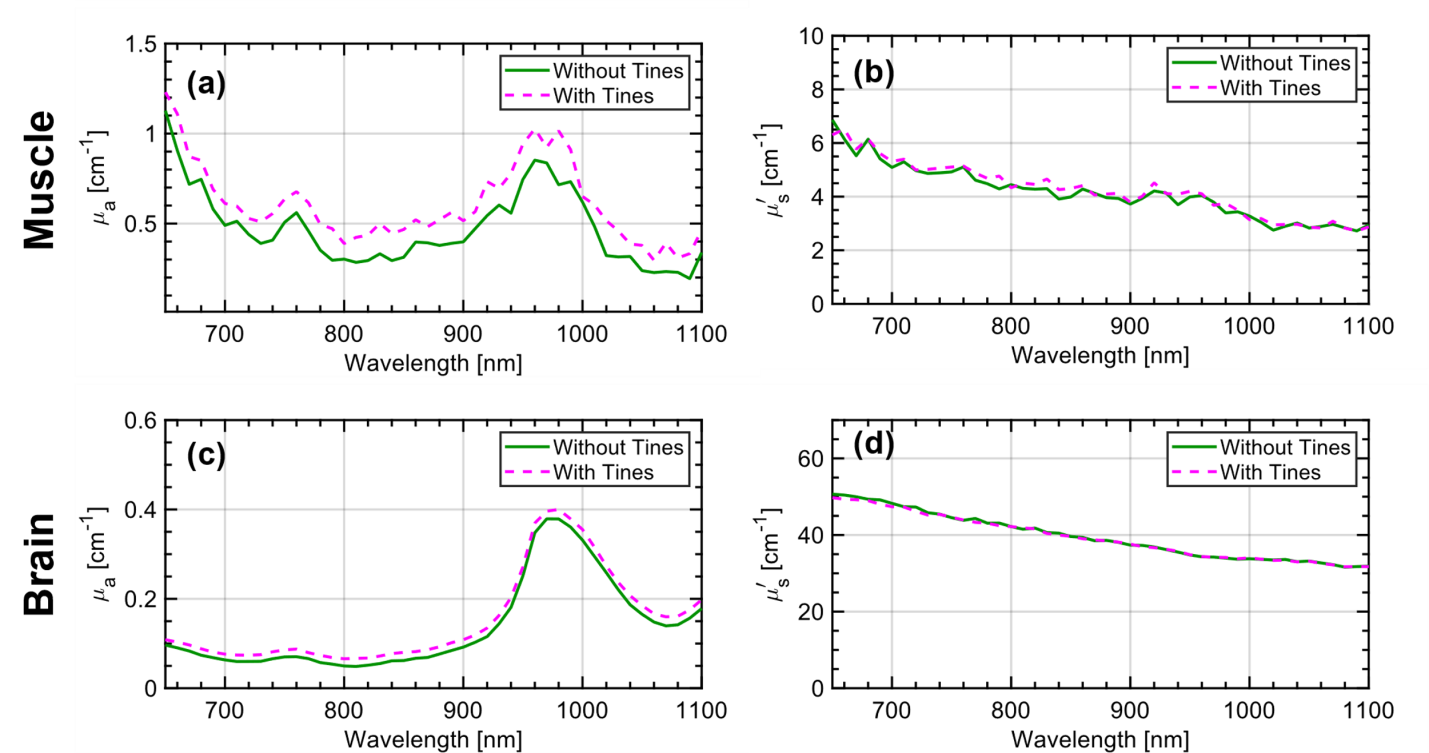


**Supplementary Figure 1. Optical properties of tissue with and without the RFA device.** (a) and (b) are absorption and reduced scattering of bovine muscle tissue with and without the RFA device (tines) in the measurement tissue volume. (c) and (d) is the corresponding spectrum from brain tissue. A small increase in absorption is visible is both cases with no difference in the reduced scattering.

# **^Evolution of the broadband optical property spectra during ablation:^**


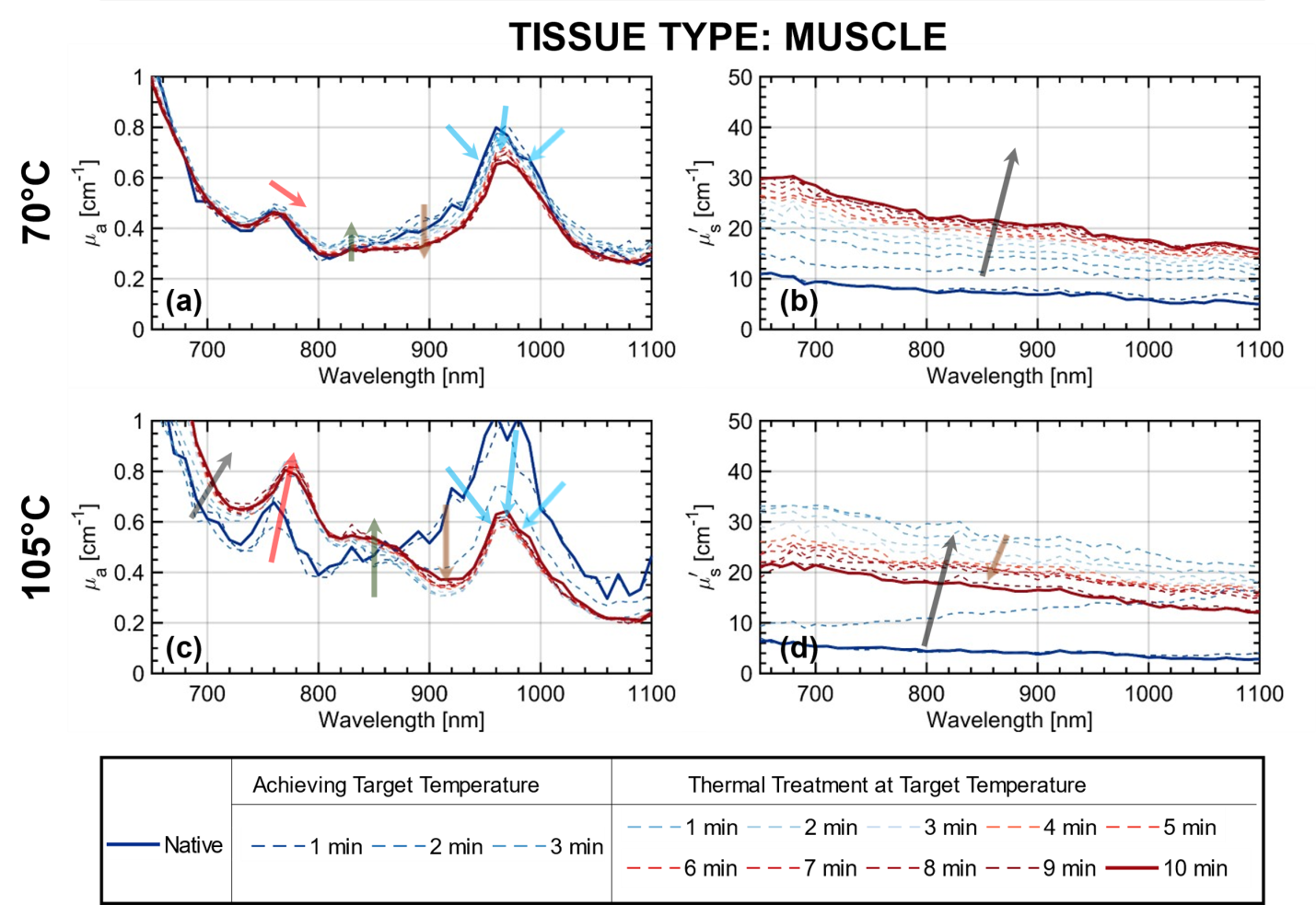


**Supplementary Figure 2. Spectral changes before, during and after ablation for muscle tissue.** Absorption and reduced scattering spectral changes before (blue), during and after (red) ablation for 70^0^C (a,b) and for 105^0^C (c,d) ablation treatment on bovine liver tissue.


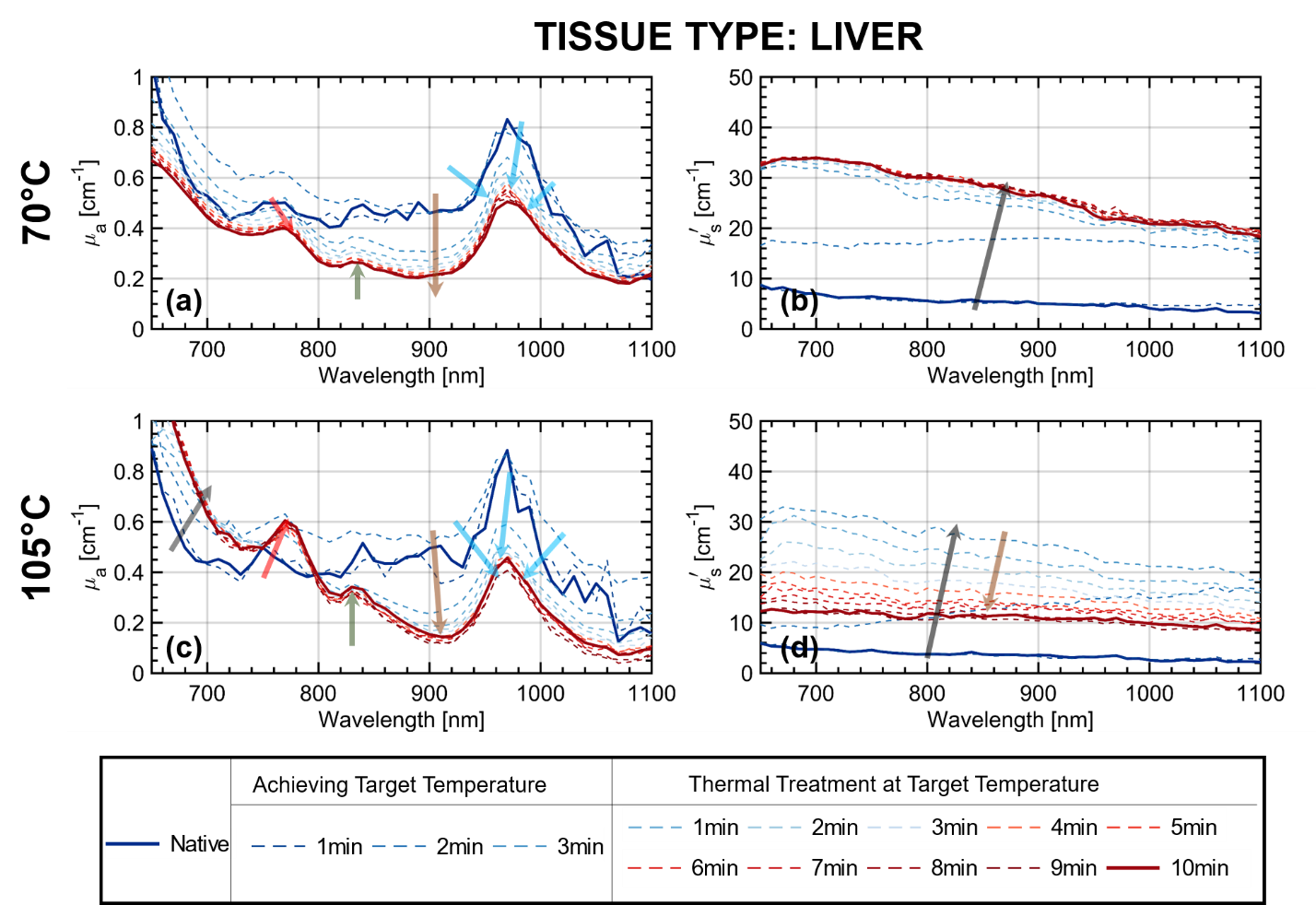


**Supplementary Figure 3. Spectral changes before, during and after ablation for liver tissue.** Absorption and reduced scattering spectral changes before (blue), during and after (red) ablation for 70°C (a,b) and for 105°C (c,d) ablation treatment on bovine muscle tissue.


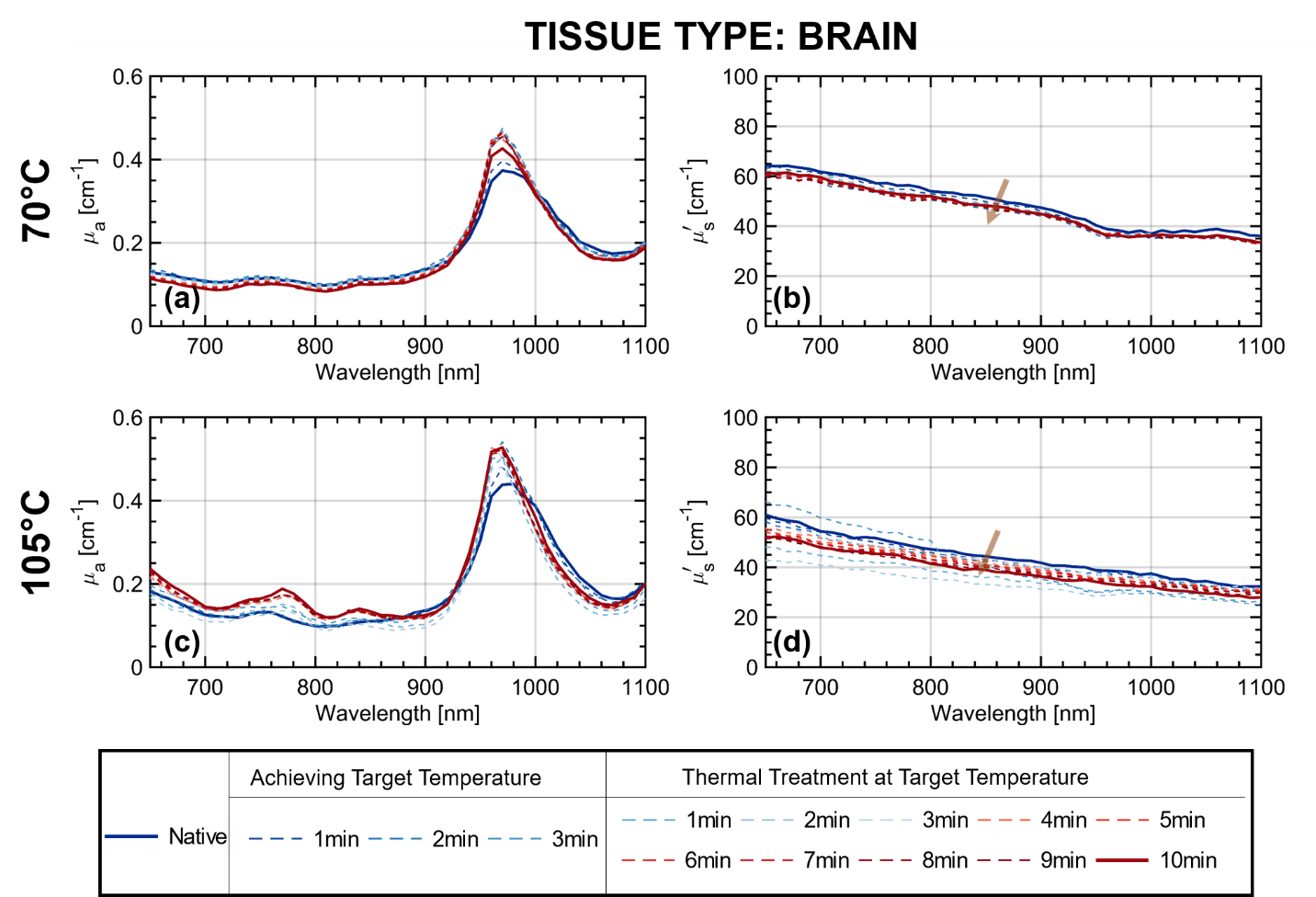


**Supplementary Figure 4. Spectral changes before, during and after ablation for brain tissue.** Absorption and reduced scattering spectral changes before (blue), during and after (red) ablation for 70°C (a,b) and for 105°C (c,d) ablation treatment on bovine brain tissue.

# **^Temporal evolution of optical properties during ablation^**


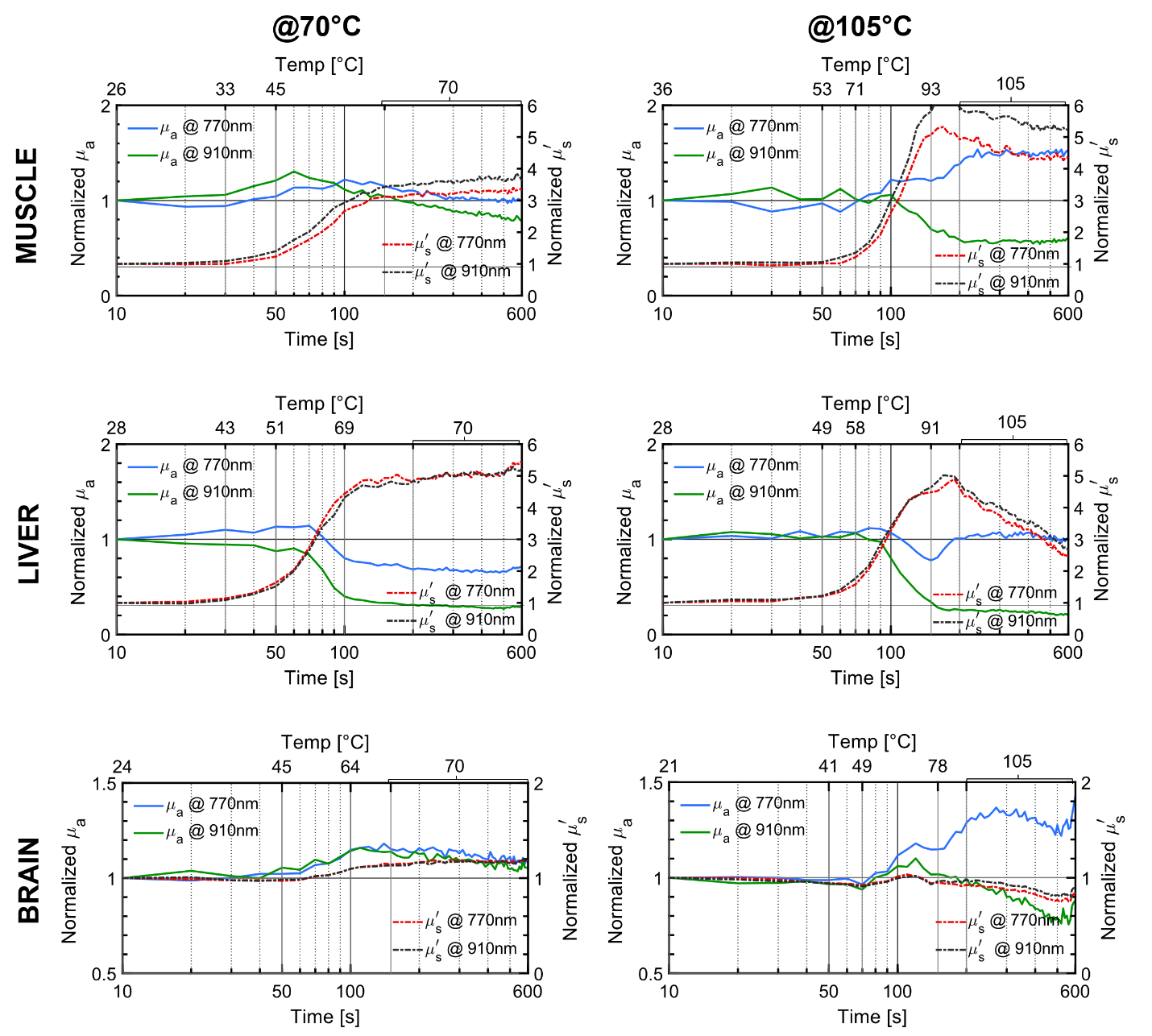


**Supplementary Figure 5. Real-time monitoring of optical properties at two wavelengths.** Changes in absorption and reduced scattering for 770 nm and 910 nm for ablation treatment with 70°C and for 105°C for bovine muscle, liver and brain respectively.
